# The digital extension of the psychotherapeutic field and the role of the body in online therapy: a grounded theory study with psychotherapists in training

**DOI:** 10.3389/fpsyg.2025.1413134

**Published:** 2025-06-25

**Authors:** Birgitta Schiller, Stella Becher-Urbaniak, Eva Wimmer, Lisa Winter, Kathrin Mörtl

**Affiliations:** ^1^Institute for Qualitative Psychotherapy Research, Department of Psychotherapy Science, Sigmund Freud Private University, Vienna, Austria; ^2^Research Department Outpatient Clinic for Adults, Department of Psychotherapy Science, Sigmund Freud Private University, Vienna, Austria; ^3^Sigmund Freud Private University Outpatient Clinic for Adults, Department of Psychotherapy Science, Sigmund Freud Private University, Vienna, Austria

**Keywords:** online therapy, body in psychotherapy, psychotherapy research, psychotherapy science, qualitative research

## Abstract

**Introduction:**

The transition to online therapy has reshaped the psychotherapeutic field, introducing novel challenges and opportunities. In the digital realm, bodily perception often appears fragmented, prompting therapists to develop new ways of maintaining presence and connection. Online therapy has thus expanded therapeutic culture through distinct experiential dynamics.

**Methods:**

This qualitative study investigated unconscious processes and bodily awareness in online therapy. Semi‑structured interviews were conducted with psychotherapy trainees at Sigmund Freud University Vienna. Data were analyzed using Grounded Theory to inductively identify central themes.

**Results:**

Findings revealed that conscious bodily awareness plays a pivotal role in online therapeutic engagement. As trainees practiced clinically in the digital setting, they constructed a new foundation for professional development. This shift influenced not only individual competencies but also fostered what can be called a digitally mediated therapeutic habitus.

**Discussion:**

While co‑presence and institutional spaces remain valuable, the evolving landscape of psychotherapy calls for reimagining traditional structures. The potential of online therapy has become increasingly evident, challenging rigid notions of therapeutic space. Further research, especially on training and professional identity formation, is essential to legitimize and integrate online therapy into the broader psychotherapeutic field.

## 1 Introduction

The importance of considering the body as a central unit in psychotherapeutic work has been underexplored in the clinical field (Shaw, 2004; Gallagher, 2005; Lemma, 2018; Dahlberg, 2019; Fuchs, 2020; Wimmer et al., 2021a; Leikert, 2022). Historically, the focus on thought and linguistic processes, such as reflection, understanding, and verbalization, has overshadowed the significance of the body (Shaw, 2004; Leikert, 2022). Although early psychotherapy authors such as Freud acknowledged the significance of the body, a comprehensive integration of the embodied experience within psychotherapy processes remained elusive (Leikert, 2022). Sensing and perceiving, vital elements of human experience, play a fundamental role in establishing therapeutic relationships and initiating processes of change (Shaw, 2004; Lemma, 2018). Embracing a multi-professional networking context is essential for psychotherapy to more fully address the role of the body, given its previous rhetorical neglect (Frommer, 1998; Tschuschke, 2017; Wimmer et al., 2021a, 2021b).

While specific therapeutic approaches focus on the body and corporeality, this paper seeks to explore the physical dimensions of online therapy without adhering to any predefined conceptual frameworks. Nevertheless, this investigation inevitably intersects with established terminologies and theoretical frameworks, as discussing corporeality often leads to rhetorical overlaps and intersections. The aim here is to consider how these physical aspects manifest in the context of online therapeutic interactions, where the absence of physical presence challenges traditional understandings of embodied experience.

The sudden outbreak of the Covid-19 pandemic highlighted the implicit interconnection of physical and psychological factors. In response to the lockdowns in the spring of 2020, therapists swiftly transitioned to online therapy. In many countries, including Austria, the online setting was not recognized as a space for psychotherapeutic treatment because psychotherapy was thus defined as an interaction between two (or more) people who are present in the same room (Eichenberg, 2021). Consequently, there was insufficient preparation and training for most psychotherapists on how to effectively conduct online therapy. Therapists faced the challenges of adapting to the digital space with limited prior experience (Inchausti et al., 2020; MacMullin et al., 2020; Aafjes-van Doorn et al., 2021; Beck-Hiestermann et al., 2021; Békés et al., 2021, 2023b; Legerer-Bratengeyer, 2021; Stefan et al., 2021). This disruption of the traditional in-person setting provided an opportunity to examine body phenomena in psychotherapy more closely, as unconscious, natural, and non-reflective actions and processes became conscious.

During the pandemic, the closure of the Sigmund Freud University (SFU) Psychotherapeutic Outpatient Clinic for Adults in Vienna posed unique challenges for psychotherapists in training (Eichenberg et al., 2021; Schiller et al., 2024). Many of them conducted online sessions from their homes, as they had not yet established their own practice space. This detachment from the training institution increased their burden of work, but it also equipped the new generation of psychotherapists with valuable skills and competencies to navigate the digital landscape of psychotherapeutic care (Schiller et al., 2024).

The experiences of therapists and patients during the pandemic expanded the scope of therapy, making a return to old familiar structures impractical. The recognition of the potential of the online setting has expanded the therapeutic field, especially in a society that has fully embraced the digital age (Abella, 2018; Kannarkat et al., 2020; Hanley, 2021; Trub et al., 2022). Notably, this study highlights the increased importance of body awareness in the digital space. The body may seem fragmented and disembodied due to spatial separation and connection through a third medium. However, establishing a presence in this state is both feasible and essential for meaningful therapeutic relationships (Riva et al., 2014; Roesler, 2017; Geller, 2021; Nayar-Akhtar, 2021; García et al., 2022). Conscious body perception plays a crucial role in developing a therapeutic relationship online (Lemma, 2017; Paiva, 2020; Carroll, 2021; Gumz et al., 2021) and forms the basis for effective and healing factors in psychotherapy (Grawe et al., 1994).

Given the complexity of corporeality in psychotherapy and its still under-researched role, a qualitative approach was chosen. The shift to online therapy has created an estrangement from conventional in-person formats, which not only offers new insights into corporeality within digital settings but also more generally opens up broader perspectives on the role of the body in psychotherapeutic processes. This study aims to investigate how psychotherapists in training perceive and negotiate the meaning of the body within this alienated online setting. Instead of applying predefined theoretical frameworks, the study follows the methodology of Grounded Theory to develop concepts inductively from participants’ lived experiences (Glaser and Strauss, 2017; Charmaz and Thornberg, 2021). This methodological approach is particularly well suited to uncover latent structures and meaning-making processes in a rapidly evolving and still underexplored field of practice.

Building on this emerging body of research, the current study aims to deepen our understanding of corporeality in online psychotherapy by focusing explicitly on bodily experience and perception. While initial qualitative studies have begun to examine the challenges and dynamics of online therapy (e.g., García et al., 2022; Lemma, 2023), corporeal dimensions and the significance of bodily awareness for the therapeutic relationship often remain implicit. Recent qualitative research on therapists’ experiences in digital formats (e.g., Békés et al., 2023b; Lagetto et al., 2024) highlights the emotional, relational, and cognitive challenges arising from the lack of physical co-presence. Although these studies do not focus explicitly on corporeality, they underscore the particular importance of nonverbal communication in the online setting.

Therapists in training provide a particularly valuable lens for examining these issues. Their reflective stance and heightened sensitivity to new therapeutic formats offer differentiated insights into bodily therapeutic practices in digital contexts—especially under conditions where institutional structures and physical co-presence are lacking (Schiller et al., 2024). A qualitative approach is therefore essential to capture the multilayered, situated, and dynamic processes that often remain inaccessible to standardized, quantitative designs.

The aim of this study is to explore how psychotherapists in training experience and make sense of corporeality in online therapy settings. The central research questions guiding this study are: (1) How do early-career psychotherapists perceive and negotiate the role of the body in the absence of physical co-presence during digitally mediated therapeutic encounters? (2) How is the estrangement from the natural therapeutic setting—where two bodies share the same physical space—experienced, and what implications does this shift have for the psychotherapeutic field?

## 2 Materials and methods

### 2.1 Participants

Participants were therapists in training at the SFU Psychotherapeutic Outpatient Clinic for Adults in Vienna, enrolled in a psychotherapy science program, who had engaged in online therapy sessions during the Covid-19 pandemic, including telephone and videophone modalities. A total of 16 interviews were conducted with psychotherapists in training at the SFU Outpatient Clinic. All participants were enrolled in the SFU’s psychotherapy science program, which follows an integrated structure combining academic and clinical components. The program includes a six-semester bachelor’s degree and a four-semester master’s degree, which run in parallel with a four-semester foundational training in psychotherapy (referred to as Propädeutikum) and a six-semester advanced clinical training within a specific psychotherapeutic modality (referred to as Fachspezifikum). After completing the first two semesters of this modality-specific training, students acquire the status of psychotherapists in training, which allows them to begin conducting psychotherapy under supervision.

At this stage—typically during the seventh semester of their studies—trainees complete their first 100 sessions with clients at the university’s outpatient clinic. Prior to entering this phase, students are required to have completed internships in social institutions and to have begun their own personal therapy, as mandated for trainee status.

Our sampling strategy followed purposive sampling, which is commonly applied in the initial stages of Grounded Theory studies (Moser and Korstjens, 2018). Based on our research scope, sampling included therapists in training who had (1) prior experience in conducting therapy in-person before transitioning to online therapy, as well as (2) therapists in training who began their therapeutic practice exclusively online during the Covid-19 pandemic. Therapists who had experience with both settings (1) could offer unique insights into the differences, challenges, and advantages of providing therapy in different formats. Therapists who started their therapeutic practice exclusively online during the pandemic (2) could shed light on their process of adaptation to the digital environment. Understanding how therapists managed to build therapeutic relationships and deliver effective interventions solely online contributes to a more comprehensive understanding of online psychotherapy.

In Grounded Theory, theoretical sampling follows initial purposive sampling in order to best contribute to the development of the emerging theory (Moser and Korstjens, 2018). In our case it became evident that interviews with therapists in training from diverse age groups and working with different psychotherapeutic modalities were necessary to gain a broader understanding of diverse therapists’ adaptation processes, so we sampled accordingly. This theoretical sampling approach aimed to enrich the understanding of online psychotherapy by capturing a wide range of experiences and perspectives within the specific context of therapists’ age and therapeutic orientations (Ligita et al., 2019; Conlon et al., 2020).

Table 1 provides an overview of the sample characteristics, detailing the demographic and clinical profiles of the participants included in this study. All therapists were at the beginning of their clinical training, conducting their first 100 sessions as psychotherapists in training. Their clinical experience at the time of the interviews ranged between one and three years. No other genders were reported aside from those listed in the table.

**Table 1:** Sample characteristics.

Interview	Gender	Age group	Psycho-therapeutic modalities	Spatial possibilities for online therapy	Living situation
1	Female	40–49	Systemic therapy	Own practice room	With 5 other people
2	Female	40–49	Individual psychology	Room at home	With one other person
3	Male	30–39	Individual psychology	Room at home	Living alone
4	Male	20–29	Individual psychology	Room at home	Living alone
5	Female	20–29	Individual psychology	Room at home and practice room	With one other person
6	Female	20–29	Psychoanalysis	Room at home and practice room	Living alone
7	Female	20–29	Psychoanalysis	Room at home	Living alone
8	Female	20–29	Person-centered therapy	Room at home	With 2 other people
9	Female	30–39	Gestalt therapy	Room at home	Living alone
10	Male	20–29	Person-centered therapy	Room at home	With 3 other people
11	Female	20–29	Cognitive behavioral therapy	N/A	Living alone
12	Male	30–39	Gestalt therapy	Room at home	With one other person
13	Female	20–29	Systemic therapy	Room at home	With 3 other people
14	Female	30–39	Systemic therapy	Own practice room	With 4 other people
15	Female	20–29	Gestalt therapy	Room at home	With one other person
16	Male	50–59	Gestalt therapy	Own practice room	N/A

Overall, the sampling approach in this study provided a diverse and representative group of therapists in training, capturing essential sociodemographic and modalities-related characteristics. The participants’ experiences and perspectives within different contexts of gender, age, therapeutic orientation, and living situations offer valuable insights into the effectiveness and challenges of online psychotherapy.

### 2.2 Setting

The study was conducted in collaboration between the SFU Outpatient Clinic and the Institute for Qualitative Psychotherapy Research at SFU. Students of psychotherapy science and psychology engaged in a research internship at the SFU Outpatient Clinic actively participated as project members under the guidance of a dedicated research coordinator (SB) located at the SFU Outpatient Clinic and the lead researcher (BS) affiliated with the Institute for Qualitative Psychotherapy Research.

### 2.3 Data collection: semi-structured interviews

The guideline for semi-structured interviews was developed to explore participants’ experiences, challenges, and perceptions related to engaging in online psychotherapy, with a particular focus on the role of the body (Schütze, 1983; Froschauer and Lueger, 2020). The interviews were conducted in the fall of 2021 with 16 psychotherapists in training. Each interview lasted between 20 and 50 min and was audio-recorded with participants’ informed consent.

The interviews followed a semi-structured and narratively oriented format. The interview guide was designed to open a space for participants to articulate their subjective experiences in their own words, while loosely structuring the conversation around four thematic entry points: (1) bodily awareness in online sessions, (2) the impact of technical factors on physical experience, (3) nonverbal communication and physical expression, and (4) changes in therapeutic space and atmosphere. A final open-ended question invited additional reflections. To ensure openness in the sense of narrative interviewing and compatibility with the Grounded Theory methodology, initial responses were given without interruption or interpretation (Schütze, 1983). Follow-up prompts were used sparingly, only to deepen emerging themes or clarify unclear passages. The structure of the guide thus served as a flexible orientation rather than a rigid framework, allowing the participants’ perspectives to guide the course and depth of each interview.

### 2.4 Data analysis: grounded theory methodology

Data analysis was conducted following the principles of Grounded Theory methodology (Kendall, 1999; Dourdouma and Mörtl, 2013; Glaser and Strauss, 2017; Charmaz and Bryant, 2019). The aim was to inductively generate conceptual categories grounded in the interview data, focusing on how therapists in training experience and interpret the role of the body in online psychotherapy. All interviews were transcribed verbatim to ensure accuracy and preserve participants’ verbal expressions.

Open Coding: In the initial phase of analysis, the entire dataset was coded using a line-by-line open coding approach (Charmaz and Bryant, 2019). Segments were identified based on thematic or perspectival shifts within the participants’ narratives. This process allowed for the emergence of initial concepts and ensured that coding remained closely tied to the participants’ language and meaning structures. The research team familiarized themselves with the data through repeated readings and engaged in constant comparison to refine and differentiate emerging codes (Charmaz and Thornberg, 2021).

Development of Second-Level Categories - Axial Coding: Building directly on the open codes, we developed a set of 16 s-level categories. These categories represent a first step of abstraction and were still closely linked to the participants’ expressions, experiences, and contextual meanings. They reflect recurrent themes across the dataset and remained grounded in the original narratives without being grouped hierarchically. These second-level categories served as a transitional analytic layer between the open codes and the more conceptual synthesis that followed (Strauss and Corbin, 1994; Kendall, 1999; Kendall, 1999; Dourdouma and Mörtl, 2013).

In a further analytical step, the research team engaged in selective coding to develop six main categories. These main categories emerged through interpretive condensation, moving beyond descriptive coding toward a higher level of abstraction. They do not serve as hierarchical containers for the second-level categories but were instead constructed through interpretive synthesis. The main categories represent broader conceptual and phenomenological patterns that cut across various second-level dimensions and respond directly to the research aim. This process culminated in the formulation of the core category: “The Digital Extension of the Psychotherapeutic Field.”

This analytic strategy is consistent with constructivist Grounded Theory, which emphasizes flexibility, reflexivity, and conceptual integration over rigid hierarchical structures (Dey, 2007; Charmaz and Bryant, 2019; Charmaz and Thornberg, 2021). Rather than seeking theoretical saturation in the classical sense, we applied the principle of theoretical sufficiency (Charmaz, 2006), assessing when analytic categories reached conceptual depth, consistency, and relevance within the scope of the study.

### 2.5 Validity and credibility

To ensure the credibility and rigor of the findings, the research team held numerous and regular group meetings to discuss the emerging concepts and categories, interpretations, and possible biases. This iterative process allowed for ongoing validation, extension and elaboration of the emerging theory, enhancing the credibility of the study (Lincoln and Guba, 1985; Elliott et al., 1999; Mayring, 2007; Steinke, 2007; Dourdouma and Mörtl, 2013; Loh, 2015; Stahl and King, 2020; Charmaz and Thornberg, 2021). The research team is rooted in the field of psychotherapy science, and all participating researchers have a psychotherapeutic background with training in various psychotherapeutic approaches. Theoretical orientations represented include Individual Psychology (IP), Psychoanalysis (PA), Systemic Therapy (ST), Cognitive Behavioral Therapy (CBT), and Gestalt Therapy (GT). Specifically, the authors’ backgrounds are as follows: BS (IP), SB (ST), LW (IP), KM (PA). In addition to the psychotherapeutic expertise, one of the authors, EW, comes from a sociological background, which allows for the integration of sociological aspects into the analysis, highlighting relevant intersections with psychotherapy. An ethic approval was granted from the Ethics Board of the Sigmund Freud University. Informed consent was obtained from all participants before participating in the study, and they were informed of their right to withdraw at any time without repercussions.

By providing additional details on the research design, data collection, and analysis procedures, this elaborate methodology section aims to offer a more comprehensive understanding of the study’s approach and enhance the transparency and credibility of the research.

## 3 Results

The following results are structured across three analytical levels: open coding (initial codes), axial coding (second-level categories), and selective coding (main categories), culminating in the development of the core category. During the initial phase of open coding, a total of 3,008 codes were generated. Through a process of analytical reduction and refinement, this number was condensed to 2,369 codes. In the iterative process between open and axial coding, 16 s-level categories were developed. These axial categories reflect recurring themes and meaning patterns across the dataset and served as the basis for further theoretical condensation.

In a further analytical step, six main categories were constructed. These emerged not through a hierarchical grouping of second-level categories, but through interpretive synthesis based on recurring conceptual tensions and phenomena that cut across the data. As such, second-level categories informed the main categories, but are not directly mapped onto them. This reflects a flexible approach in line with constructivist Grounded Theory (Charmaz, 2006; Dey, 2007).

An overview of the developed category structure—including the core category, six main categories (selective coding), and 16 s-level categories (axial coding)—is presented in Table 2.

**Table 2:** Category structure identified through grounded theory analysis.

Level	Category
Core category	The digital extension of the psychotherapeutic field
Main categories	Without presence, there are only “talking heads”
	The focusless realm and the invisible space
	The lost physical space and the absence of movement
	The paradox of closeness and distance
	Dissolution of psychotherapeutic culture - from flexibility to ambivalence
	New structures for a new setting and learning how to become a psychotherapist
Second-level categories	Adaptation
	Body awareness and self-care
	Closeness and distance
	Containment (Transference, countertransference, relationship)
	Distraction
	Ethics and ethical reflections
	Flexibility and structure
	Interventions and diagnosis
	Online therapy as intervention
	Presence
	Professional role
	Rituals
	Room and space
	Sensory perception and atmosphere
	Technology
	Visible and invisible body

Second-level categories span across and inform multiple main categories. They were not grouped hierarchically but served as a conceptual resource during interpretive synthesis.

To ground the emerging theory, the following section provides an in-depth presentation of the six main categories. Building on this, the function and significance of the core category are elaborated in relation to the role of corporeality in psychotherapy. While the increasing digitalization through online therapy forms the starting point and focus of this study, it simultaneously highlights the need for a more profound engagement with the body in psychotherapeutic practice.

### 3.1 Without presence, there are only “talking heads”

With the feeling of presence, a connection can be created despite spatial separation (Riva et al., 2014; Roesler, 2017; Geller, 2021; Nayar-Akhtar, 2021). Participants report that they physically feel that resonance can trigger a now-moment. It could also be found in the analysis of the interviews that the third medium is sometimes completely forgotten when this strong connection can be established. To be emotionally reached is therefore possible, even without direct physical presence. If this level of communication is impossible, the exchange remains on the level of “talking heads,” as one participant puts it.

Processes of mental catharsis of patients can be especially excessively tiring for treatment providers because there is no physical base to the bond which could trigger clinical change. Therefore, the interviewed therapists in training must be much more active in the online setting than they would be on-site. They share feelings and additionally ask clients how and where they perceive bodily sensations.

“*When it comes to feelings, I invite my clients to describe them, to locate them in the body. Where is the fear right now? So that they simply describe body sensations somehow. I try to report what it does to me when they tell me something. It really gives me goosebumps. I think that is an opportunity to get into this exchange, which I certainly also carry into my face-to-face therapy, because online therapy also opened up many new possibilities*.” (Interview 14)

Through the direct and permanent verbalization of physical processes, it is possible to enter a shared perceived bond. Furthermore, for the trainees, speaking pauses are hard to endure. The clients are absent and it is hard to determine what is happening in the other space. In the online therapy settings, the desire for physical connection - such as holding a patient – may arise (which usually does not happen in an in-person setting), and offering water or a handkerchief must be tolerated by the trainees without being able to act upon these impulses. But in some cases, patients’ emotions can be contained by shifting one’s posture and consciously changing the voice:

*“I had an experience at the very beginning where I thought ‘well shit, why online’. I didn’t know the client very well, and she slipped into a crisis on the screen. I have to say that not being physically present made me feel uneasy. I can intervene in a completely different way on-site than when she escalates on the screen and breaks down crying, and I can no longer see her on the screen, and I don’t know what is happening on the other site. […] I went in, believe it or not, with a certain attitude, tone of voice, and managed to calm the patient down.”* (Interview 16)

It is repeatedly reported by the therapists in training that a connection to clients can be established, although a lot is perceived to be missing, and they perceive it as a different kind of treatment.

*“Well, I cannot see the hands and the whole lower half of the body, so there is a huge part gone. It has such a ‘talking heads’ feeling. You are much more in the brain. I have the feeling that a gut connection is missing that you have with the person. However, the heart connection is not missing, so empathy and the feeling of how the other person is doing don’t disappear online.”* (Interview 1)

Another crucial factor of physicality in the online setting that is reported by the participants is that perceptions of hearing and seeing are brought more into focus in the online space. Often these are disrupted by limited internet connection, delayed sound, or poor image quality. Therefore, it is not always possible for the therapists to determine the emotional state of the counterpart. Delays in connection hinder conversational flow and necessitate additional effort to compensate. However, this leads to a more focused work from the therapists in training.

*“If the voice sounds fresh or cheerful, or when I think I’m smiling right now, and the patient cannot see that on the phone. I think it’s important that he or she knows it, then I say that, too.”* (Interview 2)

Not only is the content of what is spoken perceived with more attention, but also every nuance of voice pitch, rate of speech and variations are highlighted. Moreover, the perception of breath, especially during breaks, is helpful for the therapists in training to assess whether the person is panicking or relaxing.

*“When someone is sad or crying, there is often such a tremor in their voice. Yes, because when they cry, there is a noise lift or something at some point. The voice gets louder even when someone is angry. But not always when angry. Sometimes you are also louder when you are happy.”* (Interview 14).

### 3.2 The focusless realm and the invisible space

For the therapists in training, the body seems fragmented in the online setting due to the limited view. Therefore, what *is* visible becomes even more essential to create unity. This especially means the face and facial expressions. Thus, for the trainees the first thing that happens in their online therapy sessions is to seek the patient’s visage.

*“I get the impression that I’m not there with the client because I’m just looking past the client and not directly into their eyes. And that is much more difficult to control. There are moments in the presence where a phase comes where eye contact would be just too much, for example. Too confrontational. The clients need a time where they can look on the side – and that’s perfectly okay. To control that through an online portal —when to look, when to look away—is practically impossible because it’s not there anyway.”* (Interview 10)

The therapists report that it is strange to know that you are not looking directly at the other person. In couples therapy, person speaking is not always in focus. The couple’s sitting position may be unusual because there are positioning challenges due to the camera angle. As a result, a close sitting position may be forced upon them.

This distortion becomes even more pronounced in group settings, where they no longer know who is facing whom or what dynamics are unfolding. Side comments are entirely lost in the online format. Natural processes, such as gestures to show that you want to join the discussion, are irrelevant. Some elements are omitted in two-way dialogs, such as letting one’s view wander. This action has either the function of contemplation or distancing in order not to be at the center of a direct gaze. In the online setting, this is not possible, and spellbound tunnel-vision or concentrated stiffness develops over time.

*“[…] as if you now look directly into each other’s eyes, which of course, would be in the case that I look at the camera again and not at her. Physically I think it affects everybody differently, but it’s a stiffness, I think, that tends to emerge – stiffness now taken in a complex way, so stiffness both from the inner costume and the physical costume.”* (Interview 5)

Furthermore, the self-view in online settings leads to irritations even though it seems that the self-view is also a reassurance of the other person’s perception. Therefore, depending on the software, it cannot be always switched off or covered. What is visible in this view, especially the background, is diligently curated by the therapists in training. To get into a professional role, they dress fully and appropriately even the lower half of the body, which is not visible.

Regarding the sitting position, it is important for the therapists in training to sit upright yet comfortably. Except for occasionally being barefoot or cross-legged, there are only few variations they would not do in practice. Clients, however, are lying in bed or on the couch, looking down from above, leaning on elbows at the cell phone, or barely visible, many new patients had never attended an in-person session before. It was reported by the interviewees that it is impossible for them to assess how they would move, enter the room, shake hands or sit. Everything that happens in the non-visible room outside the view of the camera remains hidden: the nervous movement of the hands or rocking of the feet, a limp posture or possible injuries are unnoticed. For many, it was a surprise to meet the patients in-person at the SFU Outpatient Clinic after online treatment had been going on for some time.

*“[…] the whole physical aspect, the posture – I have now seen a client for the very first time again at the Outpatient Clinic, the first time in my presence, and have noticed that she is extremely restless. She then commented on that herself at the end, that she was constantly changing her posture and was very restless, and of course, I can pick up on that, but I honestly didn’t notice that at all, although she apparently also did that at home.”* (Interview 9)

### 3.3 The lost physical space and the absence of movement

Mid-career trainees rarely have their own private practice. They begin their clinical work at the SFU Outpatient Clinic, completing a mandatory part of their training. Finding safe practice space during lockdown was difficult. Therefore, most online therapy sessions were conducted from the trainees’ homes. Many of them live in shared apartments, with their partner and pets or still with their parents. In the interviews, they report the enormous efforts it took to find a quiet, professional room.

If working from home office was not possible, the therapists in training got creative and used premises such as: an old dance studio, unoccupied and empty apartments or abandoned university spaces.

With an online setting there is also no more arrival and departure. Entering and leaving a professional space are otherwise unconscious actions, which allow to step into the therapist’s role. When this is not physically possible, internal adaptations must occur. The blending of the private and the professional realms is especially challenging for the trainees, as they are not yet as established in their professional roles as their more experienced colleagues.

“I now live with my partner and our dog in a one-room apartment, which means we always have to coordinate very well because of data protection. It just doesn’t work that he is there when I’m working. We also have a balcony, but when it rains, it’s stupid. I now have another practice room I’m renovating, so I can go there with my laptop to work. I also have the journey to and from now because I drive to this room. I think that the boundaries are a bit more blurred, if you do it in your own home and then just have a quick coffee and then quickly go out with the dog during the break.” (Interview 6)

Another challenging part are distractions during sessions, including noises from roommates, pets seeking attention, a passing ambulance or construction in adjoining rooms. One therapist in training comments that distractions always happen, but they occur far more frequently in one’s home than in practice on-site.

Spatial separation during online therapy also makes shared sensory perceptions, such as sensing the room’s temperature or smell, impossible. The software used for the therapy sessions eliminates most of the background noise in the online transmissions which for some therapists in training and their clients leads to misunderstandings. However, the horizon of possible topics in therapy is also expanding, when clients are sitting on the balcony, in the car, in the garden or going for a walk.

“For example, if I’m talking to a patient and he or she is going for a walk, and I hear the birds chirping, that does something to me. Then the overall mood is perhaps more positive or more relaxed. And vice versa, if that is not so or if the voice sounds, for example, completely different than usual, then it is noticeable. I try to address that when it’s appropriate, when it strikes me, both positively and negatively.” (Interview 2)

Therefore, clients have more freedom to shape the location of the therapeutic session and more power over it which leads to discussions in the sessions about the protected setting. Otherwise, the therapists in training observe that the clients’ movement sometimes could loosen restrictions and make talking easier.

### 3.4 The paradox of closeness and distance

Closeness in online settings can be generated primarily through the presence phenomenon, which can be established in the same way as in the in-person setting by transference and countertransference. If this level of relation fails, then the treatment becomes more cognitive, and the body is left out. It can occur - as the therapists in training report - that patients encourage this, due to their problematic relationship with their bodies, e.g., eating disorders or body dysphoria.

*“I feel it most because the body is just missing now. For example, I have a patient with anxiety disorder who also has an eating disorder, and with her, it is interesting because she is quite occupied with her body. For her, it is very pleasant that you can now only see her face. So, the body is taken out of the therapy, and for her, that is something pleasant, but of course, it’s just something that’s missing.”* (Interview 6)

In addition, online treatments can have counterproductive-indicative effects for people with computer/gaming and/or media addictions. This enables putting one’s own body or physicality generally in the background or even hiding it. Therapists in training must consciously reflect on this relation, often struggling in this balancing act, as too much integration of the body can increase defensiveness. For many patients the online setting is perceived as a safe space. They feel able to interrupt the session with one click which makes it possible to discuss topics that are not (yet) possible to discuss in-person.

*“Yes, that not everything is immediately so incredibly intimate, and someone is sitting a meter in front of me looking at you or something, but that there is a certain barrier, which is often interpreted negatively for us as therapists. Which I also find disturbing, but I believe that this is very helpful for many patients at the beginning and that they find it easier to talk about unpleasant topics, such as sexuality or any taboo topics.”* (Interview 7)

Therapists in training use this distance to gain their first experiences exploring and improving their own way of practice. However, it turns out that most of the participants prefer in-person settings and that clinical work is only then gratifying.

*“It [in-person therapy] gets under your skin more, gets to you more and has more direct access to you. If you are not used to that […], it’s very quickly, suddenly, overwhelming. […] I am not a friend of the online setting simply for these reasons. I think the work is very limited by it. As a therapist, you have to be able to get involved with all the feelings that come along when you are in the same room, with this intimacy, with this privacy that is there, with the closeness that is also there. I think it is only then that psychotherapy has effect. And if you can do it well, and by now, I think I can do it a little better, only then is it really fun.”* (Interview 4)

For some patients, the therapists in training report that their own home space with its familiar atmosphere represents a protected place. For others who live with other people, the factor of conducting therapy at home becomes a particular obstacle. For example: roommates or children knocking on the door, the thought of partners eavesdropping, or parents bursting into the room when discussing sensitive topics prevent a deeper engagement.

*“I have also often witnessed that yes, they also live in a household […]. Especially when it’s family issues, that there’s just not that space to open up accordingly, especially when the people it might even affect are there, and you can only withdraw into a room […] it makes it very, very difficult to really get involved.”* (Interview 8)

The view into the private room is also ambivalent, and is experienced as expanding or intrusive, depending on the condition or perspective and the relations between therapist and client. While therapists in training prepare their background and try to hide personal things, patients are more careless or unaware. It is noticeable how patients live. This insight, about which only hypotheses can usually be formed, coincides with the character assessment. Through this expanded and shared space, new topics are possible, which can be discussed in therapy.

### 3.5 Dissolution of psychotherapeutic culture - from flexibility to ambivalence

One aspect of online therapy that was first experienced as an enormous advantage is flexibility on several levels. In terms of time, there were suddenly resources available that were not otherwise accessible. With the online setting there is a time gain from not traveling to or from the SFU Outpatient Clinic. Suddenly, it was possible to arrange appointments with clients freely and flexibly. As a result, many more new clients were taken on during this time, often scattered throughout the day and week. It happened that sessions were held late in the evening or were overdrawn. Some therapists report offers of permanent availability during crises. But this new flexibility of time was deceptive and blurred the boundaries between the professional and the private roles and had to be reconsidered - especially after the lockdowns.

*“But precisely this increased flexibility, these are also these processes which concern the dissolution of work boundaries. This increased flexibility then led me to think to myself, ‘Oh, go on, we are in a good process right now, so we’ll add another hour so that it will work out’ […]. There was a bit of a gray area with my time resources.”* (Interview 3)

In conjunction with time, space also becomes flexible for the therapists in training. They see advantages of accepting patients who live in remote regions or even from other countries and continents in an online setting. In addition, temporary injuries or minor illnesses are no longer obstacles for either the therapists or the clients. This spatial flexibility is seen as highly valuable among trainees, as it provides an extended psychotherapeutic care network. The potential loss of these resources is regarded as a striking disadvantage, especially in a global and digitally connected world.

*“[…] that spatially you can also be somewhere else. I can theoretically also be in northern Germany or wherever and can give therapy, which has advantages. For example, once I was injured and couldn’t walk. That was an advantage, that I could do therapy from home.”* (Interview 12)

During online therapy, the therapists report that patients feel safe and secure in their own space and thus behave as they would at home. Patients begin to eat, drink, smoke and more. Therapists not always know how to respond. These activities, otherwise uncommon in social space and that would never be allowed in practice rooms, disrupt the therapeutic setting in a negative as well as in a positive way.

*“This was very interesting with one patient who started eating during online therapy. This was not just once, but he would be munching on chips while we were having our sessions. […] The person is distracted by this crunching, and they cannot answer right away because then it’s always like ‘mmmmh mmmh’ and then only swallow it and so […].”* (Interview 4)

For the therapists in training, a dissolution of boundaries is perceived – especially when therapy takes place in the home office, where balancing private and professional life is complicated. Psychotherapists, in particular, need strong professional boundaries. But during online therapy, it is sometimes challenging for the trainees to get in and out of the therapeutic mindset. To achieve the shift of mindset, they reported various rituals.

*“What I’m good at is leaving things in the room. I could do that at home. That wasn’t a problem. It was just kind of weird when I walked out of the room and then took a shower right away, for example, because it’s already late in the evening. That is somehow a limit that would otherwise be there because then you have to leave the therapy room and go home, which takes at least half an hour. […] If that’s only five minutes, that’s kind of weird.”* (Interview 13)

As we have seen in the analysis of the interviews, flexibility is a tremendous asset in creating a better work-life balance. At the same time, however, it is also a limitlessness that provides little support, especially at the beginning of a career.

Individuals are still busy comparing online therapy with in-person therapy and vacillate back and forth between its advantages and disadvantages. In general, preferences toward the in-person setting were found in the study. If someone is open to online therapy, it is also evident that there is much less criticism of the technology and that the clients are more open to accepting the online setting.

### 3.6 New structures for a new setting and learning how to become a psychotherapist

Regardless of whether the attitude toward online therapy was more positive or negative, a sense of relief about returning to the SFU Outpatient Clinic was evident in all the interviews. The therapists in training report that the possibility of being in a place that can be entered as a psychotherapist, being given a structure for orientation, meeting clients in direct contact and exchanging experiences with colleagues makes acquiring the profession much more tangible. Especially at the beginning of clinical practice, the process of acquiring a professional habitus often takes place unconsciously. The role of the psychotherapist is appropriated over time through longer processes of mirroring and learning from the model. In the absence of the institution as a safe haven, the trainees developed ritualized acts to compensate for the lack of structure.

*“So I sit there, not in pajamas or so, but I sit there in work clothes. On the phone it is already possible to sit somehow comfortably, I do that already.”* (Interview 14)

Reading the protocols of previous sessions beforehand, airing the room, moving around, or other individual rituals that allowed for a process of transgression are reported by the therapists in training. In other cases, the space and the sitting posture is optimized for the use of the required technology.

*“I experience myself much more alive in [in-person] therapy and I can concentrate better. Yes, it is insanely fun. I’ve only had the first few sessions with my clients, and it’s made a huge difference for me. Honestly, it didn’t feel as real that I’m now working as a psychotherapist in training, because I was doing it from my living room and didn’t go to the Outpatient Clinic or have to deal with other therapists. So for me, it’s more real now.”* (Interview 9)

Another new practice in the effort to become a psychotherapist is also the increased need for self-care in the context of online therapy. Establishing a structure, setting boundaries in the home office, taking time before an appointment, and recovering after an online session are reported. For therapists in training, it is increasingly necessary to perform these steps in a highly conscious and reflective way.

*“Fatigue is definitely what has increased drastically in the online setting – Fatigue and also partly thoughts flying away – being absent-minded.”* (Interview 5).

As already described, the place for conducting online therapy is limited for the participants through a combination of the possibility of privacy, professional backdrop and adequate light quality, as well as a working internet connection. If such a place is provided, they need to find a way to adopt a professional posture. For the interviewees it also seems very important not to leave the area of camera coverage. Therefore, movement is hardly possible; if it is, it concerns targeted interventions with patients, like demonstrating specific interventions requiring physical participation staying within the frame of camera coverage can make the body tense. During telephone sessions without a headset, the therapists report that they experience strained posture, and that their sense of hearing is overloaded afterwards. Consequently, these therapists in training also do not want to make long phone calls or video calls to friends after work.

Social interaction is part of becoming a psychotherapist, and the missing exchange through online therapy must be considered. At the SFU Outpatient Clinic, therapists in training work within organizational structures and have access to individual guidance by experienced psychotherapists available. While online supervision and intervision did continue, the conversations in between dropped out in the online format.

### 3.7 Core category: the digital extension of the psychotherapeutic field

The Digital Extension of the Psychotherapeutic Field emerges as the primary category at the top of the second-level codes and main categories. It constitutes the fundamental phenomenon evident throughout the entire material. The term encapsulates the irrevocable transformations within the psychotherapeutic field^1^.

The therapists in training experience that psychotherapeutic work is possible online, besides some challenges, like the body, which is not present in online therapy to the same extent as in the in-person setting.

*“[…] I think it affects the psychotherapeutic relationship insanely, but I can’t say how. I think that everything is perceived, but it is perceived on an unconscious level. There’s so much going on, I can’t place it, but it [the body] certainly has an influence.”* (Interview 1)

Even though online therapy differs from other types of clinical work, and a different therapeutic relationship is established, all participants experience that therapy can be both continued and started and is indeed effective. At the first in-person sessions there are surprises, and certain assumptions must be adjusted, but a relationship has already been established.

During online therapy, listening is intensified, and trainees report to be much more active connecting and resonating with the clients and thus establishing a sense of presence. Even if an increased effort is needed, the adjustment is made quickly and unconsciously.

*“I think that we, humans, all work similarly – by getting used to the machine. Because you’ve got the fears – you’ve seen through experience through trial and error, you’ve seen it’s not that bad, and you can do quite well.”* (Interview 16)

In particular, the perception of physicality plays an important role for the therapists in training because working with the body online is more intensive and extended than in the in-person setting. Otherwise unnoticeable dynamics and processes are consciously perceived, even if they cannot be attributed entirely. Interventions, such as asking about body sensations or feedback on how they are experienced, are reported by all therapists in training regardless of which modality they adhere to.

*“I can only now say many things based on the contrast with online therapy because only then did I notice many things that were so self-evident before.”* (Interview 3)

This increased awareness of physicality is now carried over by the therapists in training into the in-person setting. The demands may be different, but their experience has improved their further clinical activity.

The therapeutic field has therefore been expanded, and the shift to online therapy has changed the psychotherapeutic culture. Questions of healthcare provision, socioeconomic relevance, availability, and reasonableness are asked in the interviews. Discussions arise, including which platforms to use, how to protect the online space and how to charge for therapeutic sessions. The therapists in training also have to learn how to use the technical applications. Clinically relevant questions of execution are being asked: How are colleagues applying an “empty chair”- work or a “family systemic constellation”- work, and what indications are given to continue psychotherapy online?

*“I think it would be cool to exchange ideas with colleagues about what different interventions they use and whether it is more difficult online. I think that can definitely be incorporated into the training as well.”* (Interview 16)

Many of them worked in an online setting from the beginning as their first clinical experience. They are for the most part technically versed and use the digital resources offered the same way as their clients. Furthermore, they all voice the demand for a more in-depth examination of online therapy. Their preference is, nonetheless, direct and close contact in a physical space. However, in their opinion, the possibility of working online is essential in the modern age, where people are globally connected and much of the social life already takes place online. Through these new possibilities this pioneering psychotherapeutic field is auspicious.

## 4 Discussion

Undoubtedly, this study’s focus on exploring the body in online therapy yielded valuable insights into otherwise unconscious processes and dynamics in a detached setting. Beyond the specific intention of this investigation, therapists’ experiences in supervised training highlight the overarching significance of the body, and embodiment, in the context of online therapy (Schiller et al., 2024).

In the realm of social media and virtual realities, the body appears fragmented, with control over its visibility and use being distinctly different (Lemma, 2017, 2018, 2023; Paiva, 2020; Carroll, 2021; Cataldo et al., 2021; Gumz et al., 2021; García et al., 2022). It is crucial for psychotherapists to remember that the body is always present, even in the online realm, and its absence becomes evident when the sense of presence cannot be established. Without a genuine feeling of presence, neither a containing therapeutic bond nor an effective therapeutic process can be initiated. Scholars have long examined this mechanism in the context of online psychotherapy (Riva et al., 2014; Roesler, 2017; Geller, 2021; Nayar-Akhtar, 2021).

The massive shift to digital formats for psychotherapeutic care in 2020 significantly contributed to expanding research in this setting. Initial doubts about the use of online portals were replaced with surprising euphoria about the possibilities they offered. Clinical work continued successfully, providing evidence for the efficacy of online therapy (Humer et al., 2020; MacMullin et al., 2020; Beck-Hiestermann et al., 2021; Merchant, 2021; Probst et al., 2021; Stefan et al., 2021; Eichenberg et al., 2022; Jesser et al., 2022; Békés et al., 2023a, 2023b; Winter et al., 2023). However, it became apparent that online therapy operates differently from traditional in-person therapy, and comparisons between the two are commonly made, with the preference often leaning toward in-person sessions (Békés et al., 2021; Leukhardt et al., 2021; Giordano et al., 2022).

Nevertheless, it is essential to recognize online therapy as a distinct form of psychotherapeutic practice rather than merely a less valuable alternative to in-person therapy (MacMullin et al., 2020; Mitchell, 2020; Chi et al., 2022; Békés et al., 2023a, 2023b; von Below et al., 2023; Lagetto et al., 2024). The transition to online therapy has brought about an upheaval that has permanently altered the field of health professions. The opportunities and potentials that emerged cannot be ignored. Particularly for a generation of newly trained psychotherapists who have been exposed to digital advancements from the outset of their professionalization (Day and Thomas-Anttila, 2021). It is important to note that all participants in this study were at the very beginning of their clinical practice, conducting their first sessions as psychotherapists in training under supervision. Their clinical experience ranged between one and three years. This early stage of professional development may have shaped the way participants perceived and negotiated bodily experience in the online setting. The absence of institutional structures and physical spaces—often central for developing a professional therapeutic habitus—was experienced with particular intensity during this formative phase. As such, the reflections captured in this study do not only provide insight into the therapeutic use of the body in online formats but also into how psychotherapists learn to embody their role under the conditions of digital transformation (Schiller et al., 2024). Despite the ambivalence surrounding online therapy, it represents an expanded resource for clinical work (Békés and van Aafjes- Doorn, 2020; Aafjes-van Doorn et al., 2021; Giordano et al., 2022; von Below et al., 2023).

The limitations and possibilities of online therapy warrant further exploration through international research. The progressive development of digital technologies and their increased integration into everyday life, elevate the importance of online therapy as a subject of investigation (Eichenberg et al., 2021; Lemma, 2023; Winter et al., 2023).

Moreover, in-depth reflection and discourse within the psychotherapeutic community are necessary concerning clinical implications. For instance, some patients, including those with social phobia or addictions, may use online therapy to maintain resistance or avoid addressing certain issues related to their bodies (Lemma, 2017, 2023; Agosta, 2019; Paiva, 2020; Adelman, 2021; Ehrlich, 2021; Chew-Helbig, 2022; von Below et al., 2023). On the other hand, online therapy might serve as a more approachable starting point for engaging in therapeutic processes and establishing a therapeutic relationship. However, therapists face the dilemma of whether therapeutic care should be allowed to proceed with this physical distance, knowing that the required closeness cannot be adequately established in the presence of such defense mechanisms (Lemma, 2017, 2023; Russell, 2018; Wajda et al., 2022; Laczkovics et al., 2023; von Below et al., 2023).

A dilemma of a harmful or harmless third medium extends to psychotherapists, especially trainees, who require the physical structures, spaces, and therapeutic fields with their unique atmosphere, aesthetics, and interpersonal dynamics to develop a professional therapeutic habitus (Rønnestad et al., 2019; Day and Thomas-Anttila, 2021; Wimmer and Wagner, 2021; Schiller et al., 2024). The transition to providing therapy from one’s own home challenges the extent to which therapists can adapt their roles, necessitating increased effort in achieving professionalization. Nevertheless, therapists in training and their patients have both acknowledged the potential of online therapy, demanding further in-depth research and development in education (Day and Thomas-Anttila, 2021; Trub et al., 2022; Békés et al., 2023a, 2023b). In a digitally evolving world, such demands do not appear unusual (Abella, 2018; Kannarkat et al., 2020; Hanley, 2021; Thomas et al., 2022).

In conclusion, our study’s findings underscore the critical role of the body and corporeality in online therapy. The shift to online formats has expanded the possibilities and resources for clinical work, necessitating further research and adaptation in the field of psychotherapy which are essential to harness its full potential and address the changing landscape of psychotherapeutic care.

## 5 Limitations

This study offers valuable insights into the role of the body in online psychotherapy, particularly from the perspective of therapists in training. However, several limitations need to be considered when interpreting the findings. First, the sample consisted exclusively of psychotherapists in training from a specific institutional context, which may limit the transferability of the results to other clinical populations or professional settings. Furthermore, while theoretical sampling is an integral part of the Grounded Theory methodology (Ligita et al., 2019; Conlon et al., 2020), the current dataset represents only an initial stage in a broader research process and does not yet capture a full range of variation—especially in relation to patients’ perspectives.

Theoretical sampling considerations also suggested that incorporating additional perspectives—such as those of patients, as well as relatives or roommates of both patients and therapists—could have further contributed to the development of the emerging theory on online psychotherapy. However, due to the scope of the research design and the available resources, this study was limited to the experiences of psychotherapists in training and did not include these additional participant groups.

Second, the study relied on self-reported experiences gathered through semi-structured interviews, which, although well-suited for an in-depth exploration of subjective meaning-making processes, may be influenced by social desirability or retrospective bias. The exclusive focus on therapists’ perspectives also means that the co-constructed dynamics of the therapeutic encounter remain only partially illuminated.

Moreover, while the Grounded Theory approach allows for inductive category development grounded in empirical data, the findings are necessarily situated within the specific socio-cultural and temporal context of the study. The data were collected at the beginning of the transition to online therapy during the COVID-19 pandemic, at a time when most therapists had to adopt digital formats out of necessity rather than choice. Since then, online psychotherapy has become increasingly normalized, and it is possible that therapists currently working in this modality are more likely to be genuinely open to it. As such, the perceptions and experiences documented in this study may differ from those emerging in more recent phases of digital integration.

Future research should aim to extend the sample to include international participants and patients, allowing for comparative analyses that account for cultural and systemic differences. Incorporating other methodological approaches, including mixed methods designs or longitudinal studies, could further enhance our understanding of corporeality in digital therapeutic contexts.

## Data Availability

The raw data supporting the conclusions of this article will be made available by the authors, without undue reservation.

## Footnotes

^1^The term ‘psychotherapeutic field’ is used here in the sense of Bourdieu’s concept of a structured social space, where various actors—therapists, clients, and institutions—interact and compete for positions, shaping the rules and practices within psychotherapy (Bourdieu et al., 1999; Kieserling, 2008; Bourdieu, 2014).
